# Metformin improves circulating endothelial cells and endothelial progenitor cells in type 1 diabetes: MERIT study

**DOI:** 10.1186/s12933-016-0413-6

**Published:** 2016-08-26

**Authors:** Fahad W. Ahmed, Rachel Rider, Michael Glanville, Kilimangalam Narayanan, Salman Razvi, Jolanta U. Weaver

**Affiliations:** 1 Department of Diabetes, Queen Elizabeth Hospital, Gateshead, UK; 2 Institute of Cellular Medicine, Newcastle University, Framlington Place, Newcastle, NE2 4HH UK; 3 Institute of Genetic Medicine, Newcastle University, Newcastle upon Tyne, UK

## Abstract

**Background:**

Type 1 diabetes is associated with increased cardiovascular disease (CVD). Decreased endothelial progenitor cells (EPCs) number plays a pivotal role in reduced endothelial repair and development of CVD. We aimed to determine if cardioprotective effect of metformin is mediated by increasing circulating endothelial progenitor cells (cEPCs), pro-angiogenic cells (PACs) and decreasing circulating endothelial cells (cECs) count whilst maintaining unchanged glycemic control.

**Methods:**

This study was an open label and parallel standard treatment study. Twenty-three type 1 diabetes patients without overt CVD were treated with metformin for 8 weeks (treatment group-TG). They were matched with nine type 1 diabetes patients on standard treatment (SG) and 23 age- and sex-matched healthy volunteers (HC). Insulin dose was adjusted to keep unchanged glycaemic control. cEPCs and cECs counts were determined by flow cytometry using surface markers CD45^dim^CD34^+^VEGFR-2^+^ and CD45^dim^CD133^−^CD34^+^CD144^+^ respectively. Peripheral blood mononuclear cells were cultured to assess changes in PACs number, function and colony forming units (CFU-Hill’s colonies).

**Results:**

At baseline TG had lower cEPCs, PACs, CFU-Hills’ colonies and PACs adhesion versus HC (p < 0.001-all variables) and higher cECs versus HC (p = 0.03). Metformin improved cEPCs, PACs, CFU-Hill’s colonies number, cECs and PACs adhesion (p < 0.05-all variables) to levels seen in HC whilst HbA1c (one-way ANOVA p = 0.78) and glucose variability (average glucose, blood glucose standard deviation, mean amplitude of glycaemic excursion, continuous overall net glycaemic action and area under curve) remained unchanged. No changes were seen in any variables in SG. There was an inverse correlation between CFU-Hill’s colonies with cECs.

**Conclusions:**

Metformin has potential cardio-protective effect through improving cEPCs, CFU-Hill’s colonies, cECs, PACs count and function independently of hypoglycaemic effect. This finding needs to be confirmed by long term cardiovascular outcome studies in type 1 diabetes.

*Trial registration* ISRCTN26092132

**Electronic supplementary material:**

The online version of this article (doi:10.1186/s12933-016-0413-6) contains supplementary material, which is available to authorized users.

## Background

Type 1 diabetes mellitus is characterised by an increased risk of cardiovascular disease (CVD) compared with the non-diabetic population [1, 2]. The life expectancy of adults at age of 20 with type 1 diabetes is reduced by up to 13 years with CVD being the leading cause of premature death [3]. Even with good glycaemic control the CVD risk remains more than twice that of non-diabetic individuals [4, 5]. Despite current use of statins and ACE-inhibitors, CVD risk in type 1 diabetes remains higher than non-diabetic population. Patients with type 1 diabetes mellitus without overt CVD or diabetes-related complications have been shown to have features of endothelial dysfunction [6]. There is a need to explore newer treatment options to improve endothelial dysfunction and reduce CVD risk. Endothelial dysfunction itself results from imbalance between vascular damage and vascular repair.

Vascular damage results in the release of circulating endothelial cells (cECs) from the vascular intima. cECs are mature endothelial cells characterised by presence of endothelial cell surface markers like CD144 and absence of heamatopoetic (e.g. CD45) and progenitor cell markers (e.g. CD133). CD144 is important for maintaining endothelium integrity through cell to cell adhesion [7].

cECs are formed through detachment from vascular intima due to irreversible loss of integrity as a response to endothelial activation by mechanical stress, inflammatory cytokines, growth factors, infectious agents, lipoprotein, and oxidative stress [7, 8]. Furthermore, cECs are elevated in type 1 and type 2 diabetes [9, 10], and are a predictor of CVD events in similar high risk populations [11]. cECs count (a marker of vascular damage) is directly related to HbA1c in type 1 diabetes [9].

In response to vascular damage, vascular repair is promoted by local endothelial cells and bone-marrow derived cells, called endothelial progenitor cells (EPCs). EPCs were first described in 1997 [12]. These cells have the ability to home to the site of vascular injury, proliferate and contribute to endothelial repair [13], thereby maintaining endothelial health.

Circulating endothelial progenitor cells (cEPCs) are a heterogenous population of cells characterised by the expression of surface antigen CD34^+^, VEGFR-2+ and/or CD133+ identified by flow cytometry. CD34^+^ and CD133+ are hematopoietic stem cell markers [14, 15]. VEGFR-2 is a surface marker of endothelial lineage. Progenitor cells undergo various stages of maturation. CD133 marker is lost as cEPCs mature. Thus, more mature cEPCs are positive for CD34 and VEGFR-2 [16]. VEGFR-2 plays an important role in angiogenesis by promoting endothelial cell growth and cell permeability [17]. cEPCs predict microvascular complication in type 2 diabetes [18] and future CVD events in patients with CVD [19]. In addition cEPCs count is inversely related to HbA1c [20].

Proangiogenic cells (PACs), previously known as early EPCs are the cultured peripheral blood mononuclear cells (PBMNC) whereas colonies derived from replated PBMNC are known as colony forming units (CFU-Hill’s colonies) [21]. PACs and CFU-Hill’s colonies are reduced in both type 1 and type 2 diabetes and CFU-Hill’s colonies have been shown to predict CVD events [20–24]. PAC count is inversely related to HbA1c and much more suppressed in the presence of diabetes-related complications [23–25]. In addition, the functional capacity of cultured PACs is impaired in patients with diabetes [23].

As the outcome of CVD management using the same therapies is worse in diabetic versus nondiabetic patients [26], there is a need to identify additional treatment options and study the mechanism of action behind the cardio-protective properties. Metformin has been shown to have cardio-protective properties in type 2 diabetes, [27, 28]. In the UKPD trial [27] the incidence of myocardial infarction was reduced after a median follow-up of 10 years. Furthermore, metformin also reduced cardiac infarct size and improved endothelial function in diabetes [29, 30]. It has been shown that metformin under diabetic environment protects ECs regardless of its glycaemic effects [31]. In nondiabetic patients however, there were mixed findings regarding metformin’s cardio-protective effect. Metformin had no effect on reducing left ventricular dysfunction and re-perfusion cardiac injury in non-diabetic patients following an acute myocardial infarction and coronary arterial bypass graft [32, 33].

Thus, the data are in keeping with a cardio-protective effect of metformin in diabetes, although the underlying mechanism is unclear. Since metformin has been shown to improve endothelial function in type 1 diabetes, we hypothesised that metformin modulates cEPCs and cECs count and this cardio-protective effect is mediated beyond improving glycaemic control.

Thus the primary aim of our trial was to study if metformin improved cEPCs number in type 1 diabetes whilst, maintaining unchanged diabetic control. Secondary aims were to determine if metformin also improved cECs number, PACs number and function and CFU-Hill’s colonies.

## Methods

**Fig. 1:**
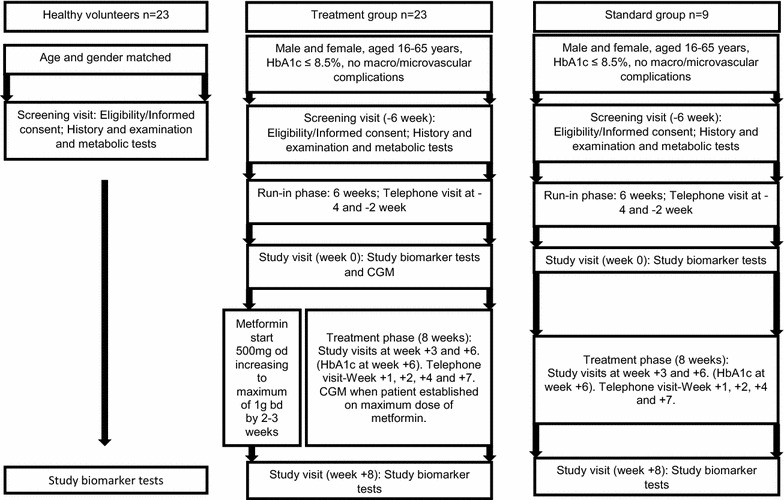
Schematic diagram illustrating MERIT study design. *CGM* continuous glucose monitoring

We recruited 23 patients with type 1 diabetes with inclusion criteria of HbA1c <8.5 % (69 mmol/mmol), absence of macrovascular disease or stage 3b renal impairment (eGFR <45 ml/min/1.73 m^2^) or active proliferative retinopathy, as the ‘treatment group’ (TG). Nine matched type 1 diabetes patients were recruited as a standard group (SG). Both, TG and SG did not have any new intervention during the trial except for metformin in TG. Patients with suspected hypoglycemia unawareness were excluded. The study protocol (Fig. 1) included a *run*-*in* phase of 6 weeks to ensure stable glucose control. Following this period metformin was given for 8 weeks to TG with a dose titrated up to a maximum of 1 g twice a day over 2–3 weeks or to highest tolerated dose. The SG underwent similar follow-up except for metformin treatment. Furthermore, the TG was compared with 23 age- and gender-matched non-diabetic healthy controls (HC). All subjects gave their written informed consent and the Local Ethics Committee approved the study. Patients with type 1 diabetes were recruited either from, Queen Elizabeth Hospital, Gateshead or Royal Victoria Infirmary, Newcastle, UK. Healthy controls were recruited from the staff from the above or students from Newcastle University, UK.

Routine laboratory investigations (full blood count, U&Es, liver function test, thyroid function test, and HbA1c), 12-lead ECG, blood pressure, weight, height and BMI were performed at baseline and at the end of the study.

We aimed for unchanged glycaemic control during the study, which was assessed by HbA1c (four times points over 14 weeks) and continuous glucose monitoring (CGM) (Ipro2- Medtronic) (minimum of 48 h) was performed in those receiving metformin to ensure unchanged glycemic control. EasyGV Version 8.8.2. R2 was used to calculate glucose variability index [34].

Peripheral EDTA blood samples were collected before and after the study from TG and SG and at baseline from HC.

### Endothelial progenitor cells

#### Flow cytometric evaluation of circulating endothelial progenitor cells (cEPCs) and circulating endothelial cells (cECs)

100 µl of whole blood [6] was incubated with 5 µl of V500 CD45 (B.D Bioscience), 20 µl of PerCP-Cy5.5 CD34 (BD Bioscience), 5 µl of PE VEGFR-2+ (R&D), 5 µl APC CD133 (Miltenyi Biotec USA), 10 µl of FITC CD144 for 30 min. Subsequently, 2 mls of pharmlyse (BD Bioscience) was used to lyse the red cells. The sample was then analysed by flow cytometry on BD FACS Canto™ II system and results by using BD FACSDiva™ software. On average 450,000 events were counted. cEPCs were defined as CD45^dim^CD34^+^VEGFR-2^+^ cells and cECs as CD45^dim^, CD133^−^, CD34^+^, and CD144^+^ events. cEPC count was expressed as % leukocytes (Intra-assay variation <8 %) and cECs as per ml of blood.

### In-vitro assays

Methods for each of in vitro assay are described in details in Additional file 1. Assays described are: (1) enumeration of proangiogenic cells (PACs); (2) Colony forming units (CFU-Hills’ colonies; and (3) PAC function: fibronectin adhesion assay.

### Statistical analysis

The results are expressed as mean ± SD unless stated otherwise. Within group (treatment or standard) comparison was evaluated by paired Student *t* test or Wilcoxon Signed Rank test depending on the distribution. Between-groups, comparison was by unpaired Student *t* test or the Mann–Whitney test. Correlation between different parameters were calculated by Pearson correlation or Spearman’s rho analysis. Multivariate regression analysis of delta changes in parameters were used to determine if independent metabolic variables predicted an improvement in cEPCs, PACs, CFU-Hill’s colonies, cECs and PACs function. One-way ANOVA was used to analyse the difference between HbA1c values. Adjustment for multiple comparisons was made by using the Bonferroni correction. Statistical significance was accepted at p < 0.05 (two-tailed significance).

As the aim of the study was to assess the effect of metformin on cEPCs in type 1 diabetes, therefore, statistical power calculation was undertaken only for the TG. Based on our pilot work and in order to reduce CVD risk in patients with type 1 diabetes, we aimed to detect a difference of 0.0021 in cEPCs (% leukocytes) in the treatment group (before and after metformin treatment), with α = 0.05 and a power of 90 %, a minimum of 20 patients were required. SPSS v21.0 (SPSS Inc, Ill) was used to perform statistical analysis.

## Results

### Clinical characteristics

Baseline characteristics of three groups are shown in Table 1. All groups were well matched for age, gender and blood pressure. TG and SG had a similar duration of diabetes (DOD), HbA1c, baseline insulin dose, lipid profile and creatinine. BMI was lower in SG in comparison to TG.

**Table 1:** Subject’s clinical and metabolic characteristics

	TG (n = 23)		p value TG V1 vs V2	HC (n = 23)	p value HC vs TG V1	SG (n = 9)		p value SG V1 vs V2	p value SG V1 vs TG V1
	TG V1	TG V2				SG V1	SG V2		
Age year	46 ± 13	–	–	46 ± 12.6	1	47.4 ± 13.6	–	–	0.8
Sex M/F n	11/12	–	–	11/12	–	5/4	–	–	–
DOD years	23 ± 13.6	–	–	–	–	23.7 ± 14.1	–	–	0.9
BMI kg/m^2^	28.7 (24-32)	29 (23-32)	>0.05	26.2 ± 4.7	0.1	23.8 (22–27)	23.7 (21.3–27.1)	0.3	<0.05
Systolic BP mmHg	125 ± 10.8	121 ± 14	0.2	119.4 ± 9	0.2	132.8 ± 6.2	130.8 ± 12.1	0.7	0.05
Diastolic BP mmHg	76.2 ± 9.2	74 ± 7	0.1	75.7 ± 9	0.9	77 ± 8.2	72.9 ± 3.6	0.4	0.8
HbA1c mmol/mol	56.9 ± 10.5	55.9 ± 8.5	0.5	34.8 ± 2.9	<0.0001	58.6 ± 7.4	59 ± 9	0.7	0.6
HbA1c %	7.3 ± 0.9	7.3 ± 0.8	0.6	5.3 ± 0.3	<0.0001	7.5 ± 0.70	7.5 ± 0.8	0.6	
Insulin dose units	44 (20–69)	39 (18–66)	<0.001	–	–	52.3 ± 11	52.9 ± 11	0.5	0.4
Smoking y/e/n	4/2/17	–	–	0/0/23		2/1/6	–	–	–
Total cholesterol mmol/l	4.5 ± 0.8	4.4 ± 1	0.2	4.96 ± 0.8	0.1	4.8 ± 1.3	4.9 ± 1.4	0.8	0.7
Triglyceride mmol/l	0.9 ± 0.4	0.9 ± 0.4	0.9	1.5 ± 0.9	0.008	0.7 ± 0.32	0.7 ± 0.3	0.6	0.2
HDL-cholesterol mmol/l	1.8 ± 0.5	1.6 ± 0.4	<0.05	1.6 ± 0.4	0.1	1.9 ± 0.6	2.1 ± 0.6	0.4	0.5
Creatinine umol/l	73 (68–94)	70 (63–77)	0.01	78 (70–87)	0.3	75 (65–87)	77 (62.8–83.5)	0.7	0.7
WCC	6.4 ± 2.4	6.3 ± 2	0.7	6.3 ± 1.6	0.9	5.8 ± 1.5	5.6 ± 1.7	0.9	0.5

Values are given as mean ± SD or * median [Interquartile range (IQ)]

*kg* kilogram, *BMI* body mass index, *BP* blood pressure, *M* male, *F* female, *DOD* duration of diabetes, *Y* yes, *E* ex-smoker, *N* no, *TG V1* Pre-treatment, *TG V2* Post treatment, *SG V1* Pre-observation, *SG V2* Post observation, *WCC* White cell count

In TG, at recruitment twelve patients took aspirin and/or ACE inhibitor and/or statins in addition to insulin. No new medication other than metformin was started during the trial (except for metformin in the TG). No medication dosage was changed other than the dose of insulin and metformin. HC took no aspirin, ACE inhibitors and/or statins. Five patients in the SG took aspirin or/and ACE inhibitor and/or statins in addition to insulin. There was no difference in medication between TG and SG.

After treatment with metformin, BMI, total cholesterol, triglyceride, blood pressure and HbA1c remained unchanged. Over 14 weeks HbA1c values were as follows −6 week (56.4 ± 8.3 mmol/mol, 7.3 ± 3 %), 0 week (56.85 ± 10.5 mmol/mol, 7.3 ± 0.9 %), +6 week (56.8 ± 8.5 mmol/mol, 7.3 ± 0.8 %) and +8 week (56 ± 0.8 mmol/mol, 7.3 ± 0.8 %); one-way ANOVA, p = 0.78). The coefficient of variation of HbA1c over 14 weeks was 4.8 %. Furthermore, continuous glucose monitoring confirmed unchanged glucose control and variability (Average glucose CGM mmol/l: 9 ± 3 vs 8 ± 2.3, p = 0.17; blood glucose standard deviation: 3.3 ± 1.1 vs 3 ± 1.2, p = 0.3; mean amplitude of glycaemic excursion; 7 ± 2.7 vs 6 ± 3, p = 0.3; continuous overall net glycaemic action: 7.7 ± 2 vs 7.3 ± 2.2, p = 0.4; Total area under curve (AUC) (calculated): 12341 ± 2900 vs 11500 ± 3182, p = 0.3; AUC above limit-7.8 (CGM): 1.86 vs 1.97, p = 0.7. Insulin dose, HDL cholesterol and creatinine were significantly reduced in the TG treatment group after metformin treatment. There were no changes in any variables in SG.

### Side effects

None of the volunteers in the study suffered any side effects requiring discontinuation of metformin. Fifteen patients took the full recommended dose of metformin (1000 mg BD). One patient took 500 mg BD due to low low eGFR (46 ml/min/1.73 m^2^). Five patients had gastrointestinal side effects that required dose reduction (two patients took 500 mg TDS; two took 500 mg BD, and one took 500 mg OD). No patient suffered any major or severe episode of hypoglycaemia. Major episode of hypoglycaemia was defined as any episode of low blood sugar requiring intervention of another person to resolve the event. Severe hypoglycaemia was defined as any episode of hypoglycaemia resulting in loss of consciousness. There was no significant effect of metformin on minor hypoglycaemic events (% ≤3.9 mmol/l and area under curve 3.9 mmol/l on CGMS: 8.6 % vs 13.3 %; p = 0.2 and 0.08 vs 0.1; p = 0.5 respectively).

### Study biomarkers

**Fig. 2:**
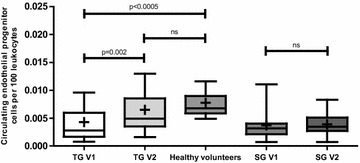
Circulating endothelial progenitor cells CD45^dim^CD34^+^ VEGFR-2^+^. Results given as per 100 leukocytes. *TG V1* treatment group pre-metformin, *TG V2* treatment group post-metformin, *SG V1* standard group pre observation, *SG V2* standard group post 8 weeks observation. *Line* denotes in each *box* as median and + in each *box* denotes mean value

Figure 2 provides a comparison of cEPCs while Table 2 provides a comparison of cECs, PACs, CFU-Hill’s colonies and PACs adhesion function between the TG, SG and HC.

**Table 2:** Indices of vascular health measured bef ore and after metformin therapy

	Treatment group (TG)		p value TG V1 vs V2	Healthy controls	p value TG V1 vs HC	p value TG V2 vs HC	Standard group (SG)		p value SG V1 vs V2	p value TG V1 vs SG V1
	V1 (Pre metformin)	V2 (Post metformin)		HC			V1	V2		
PACs per hpf	16.6 ± 8.9	28.4 ± 12.8	<0.0005*	40.3 ± 20.2	<0.0005*	0.07*	17.6 ± 12	15 ± 11	0.6	0.8
FAA per hpf	26.9 ± 21	61 ± 42	<0.0005*	67 ± 29	<0.0005*	0.15	35.9 ± 15	37 ± 16	0.9	0.1
Hill colonies per well	8.3 ± 6.8	13.8 ± 9	<0.0005*	20.57	<0.0005*	0.1*	10.4 ± 6.6	11 ± 6.2	0.8	0.5
(CECs) CD45^dim^CD34^+^CD133^−^CD144^+^ per ml^	74.4 (46.4–221)	47.6 (21.8–76.7)	<0.05*	42.6 (12.7–66)	0.03*	0.7	99.8 (59.4–210.5)	119.5 (80.5–527.5)	0.5	0.7

Values given as mean ± SD or ^ median (Interquartile range)

*PACs* proangiogenic cells, *FAA* fibronectin adhesion assay- Adhesion of PACs, cECs circulating endothelial cells, combination of CD45^dim^CD34^+^CD133^−^ and CD144^+^, *TG V1* pre-treatment, *TG V2* post treatment, *SG V1* pre-observation, *SG V2* post observation

* Results after Bonferroni correction

### Circulating endothelial progenitor cells

**Fig. 3:**
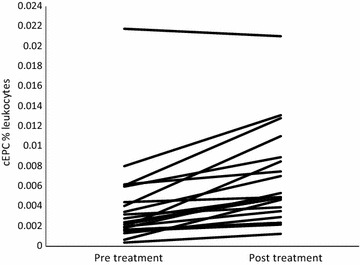
Twenty-three plots representing cEPC pre and post metformin treatment in treatment group

cEPCs count was similar in TG and the SG at baseline (p = 0.4). cEPCs (CD45^dim^CD34^+^VEGFR-2^+^) were significantly lower (60 %) in TG versus HC [Treatment group pre-metformin (TG V1 vs HC); median intraquartile range (IQ): 0.0028 (0.0016–0.006) vs 0.0068 (0.006–0.009)  % leukocytes; p < 0.0005)]. Eight weeks metformin treatment significantly increased cEPCs in TG by more than 75 % and normalised the levels of cEPCs count when compared to HC [TG V1 vs TG V2 (Treatment group post metformin); median (IQ): 0.0028 (0.0016–0.006) vs 0.005 (0.0035–0.0085) % leukocytes; p = 0.002]. In SG 8 weeks of standard follow-up did not result in any change in cEPCs count [SG V1 vs SG V2: median (IQ): 0.0032 (0.002–0.004) vs 0.0035 (0.003–0.005)  % leukocytes; p = 0.6]. Figure 3 provides before and after treatment plots for cEPC in TG.

### Circulating endothelial cells

cECs number was similar in TG and SG at baseline. However, cECs were significantly higher (74 %) in TG versus HC. Metformin treatment led to a significantly reduction of cECs (36 %) in TG. Furthermore, metformin treatment normalised cECs numbers versus HC. cECs numbers did not change after eight-week follow-up in the SG.

### Culture Assay for PACs and CFU-Hill’s colonies

PACs and CFU-Hill’s colonies numbers were similar in TG and the SG at baseline. PACs and CFU-Hill’s colonies counts were significantly lower (59 and 60 % respectively) in TG compared with HC. Eight weeks metformin treatment significantly increased PACs and CFU-Hill’s colonies in TG by 71 and 66 % respectively. Metformin treatment seemed to bring PACs and CFU-Hill’s colonies count closer to HC levels. After 8 weeks of follow-up in SG, the PACs and CFU-Hill’s colonies numbers remained unchanged.

### PACs adhesion to fibronectin

The adhesion of PACs was similar in TG and SG at baseline. The adhesion of PACs in TG was 60 % lower when compared to HC. Metformin led to a significant increase (127 %), in PACs adhesion in TG and to the level seen in HC.

The PACs number remained unchanged after eight weeks of follow-up in SG.

### Correlation

#### Univariate analysis in TG

In univariate analysis in TG, there was no correlation between changes in HbA1c, BMI, insulin dosage, total cholesterol, HDL cholesterol, LDL cholesterol, cEPCs, PACs, CFU-Hill’s colonies levels and PACs adhesion. There was an inverse correlation between changes in CFU-Hill’s colonies and cECs number (r = −0.6; p = 0.003) in TG. There was an inverse correlation between changes in PACs number and triglycerides (r = −0.6; p = 0.001) in TG.

#### Multivariate regression in TG

In multivariate regression analysis none of independent variables (changes in HbA1c, BMI, insulin dosage, LDL cholesterol) predicted changes in cEPCs, PACs number and function, CFU-Hill’s colonies and cECs.

Univariate and multivariate regression analysis are given in Additional file 1: Tables S1 and S2 respectively.

## Discussion

In this study, we have demonstrated for the first time that in patients with relatively well controlled type 1 diabetes mellitus (mean HbA1c 7.3 or 56.4 mmol/mol), metformin therapy improved markers of vascular damage (cECs) and repair (cEPCs). We believe that this study may have positive clinical implication for patients with increased CVD risk by rebalancing the emphasis in their management from limiting damage alone to also improving vascular repair. Further evidence that our patients might benefit from metformin comes from the fact that CFU-Hill’s colonies, PACs number and adhesion properties improved significantly. It is well established that CFU-Hill's colonies number are inversely related to Framingham risk score. Therefore, CFU-Hill’s colonies are yet another predictor of CVD [21]. In addition, PACs adhesion function is an important factor in cEPCs homing, cell-cell contact and transmigration events for neovascularisation and vascular repair [35]. Metformin not only improved the level of cEPCs but also brought PACs number/adhesion and CFU-Hill’s colonies closer to the HC levels.

In addition, for the first time we have shown that in TG there was an inverse correlation between changes in cECs and CFU-Hills’ colonies. This shows that changes in markers of vascular/endothelial damage are linked inversely with a marker of CVD risk (CFU-Hill’s colonies).

The additional benefit suggested by our study for patients with type 1 diabetes is that the vascular health/repair may be improved in already well-controlled patients and without a need for further improvement in glycaemic control. Patients with type 1 diabetes are currently advised to achieve HbA1c <7 % or <54 mmol/mol in order to reduce CVD events. However, this is associated with inherent risk of experiencing hypoglycaemia. The recent work by the EURODIAB Prospective Complications Study has demonstrated a U shaped association between all-cause mortality and HbA1c. That is, all-cause mortality is highest at low (5.6 %; 37.7 mmol/l) and high (11.8 %; 105.5 mmol/mol) HbA1c [36]. Thus, an additional advantage of using metformin in type 1 diabetes suggested by our study is that markers of vascular health and repair may be improved without lowering blood glucose concentrations to a tightly control HbA1c level.

Metformin has been shown to improve cEPCs in type 2 diabetes [37]. However, there was a significant improvement in HbA1c. Thus, the change in cEPCs number could have been attributed to improved diabetic control. We have shown that the effect of metformin on all vascular biomarkers studied was beyond improving diabetic control. Indeed, HbA1c and glycaemic variability remained unchanged after 8 weeks of therapy. The glucose independent mechanism behind the metformin cardio-protective effect is of particular interest as this drug has some beneficial cardiac properties in non-diabetic animals [38]. When used in non-diabetic animals, metformin improved the outcome and revascularization following surgically induced myocardial infarction and hindlimb ischemia [38, 39]. Insulin dosage was reduced significantly, but it was not correlated with changes in cEPCs number or PACs adhesion. This is interesting, as insulin has been shown to improve cEPCs number [40, 41] and function in type 2 diabetes [42, 43]. However, the improvement in cEPCs number in Fadini et al. [41] could be attributed to improvement in HbA1c. This is in contrast with Humpert et al. [40], who showed that effect of insulin on the cEPCs number is independent of HbA1c. If the former was likely, given the reduction in insulin dosage in our study, cEPCs, PACs and CFU-Hill’s colonies number and PACs adhesion should have decreased, but this is not the case. This suggests that the insulin dose reduction did not have any effect on improving cEPCs or PACs function. Reduction in insulin dose had no effect on any variable including cEPCs in univariate or multivariate analysis.

cECs are recognised markers of vascular damage. Our study showed that metformin therapy improved the cECs count in type 1 diabetes and brought it closer to the matched HC. Even though cECs improved significantly, we believe that our study did not show the full effect of metformin on cECs, as some of our patients were using cardio-protective drugs already: statins and ACE inhibitors. Indeed, in TG subjects on ACE inhibitors and or statins less reduction of cECs was observed.

Eight weeks of metformin treatment did not result in any significant change or BMI. This is in contrast with a recent study which showed that 6 months of metformin in people with type 1 diabetes resulted in the loss of nearly 2.5 kg weight when compared to placebo [30]. However, we requested that patient would not aim to improve their diabetic control whilst in the study, so it is possible explanation for the lack of weight loss. Surprisingly, HDL cholesterol levels were reduced after metformin therapy, though, were similar to the control group and remained well within the normal range. However, there was no correlation between changes in cEPCs with BMI nor HDL cholesterol in univariate analysis. Thus, in our study it seems that BMI and HDL cholesterol are not responsible for metformin’s effect on the cEPCs number. This is in contrast with available evidence where HDL cholesterol has been shown to play a role in number EPCs and ischemia induced endothelial repair [44–46]. Furthermore, the multivariate analysis also showed that change in BMI was not a predictor of cEPCs either.

Our work can be supplemented further by understanding the mechanism through which metformin improves cEPC and cECs numbers. It is established that EPC differentiation and mobilization is impaired in diabetes mellitus patients [47]. Hyperglycaemia induces endothelial cell death via suppression of SIRT1. In-vitro work has shown metformin’s effect on improved cell survival (at physiological levels) although in mouse cells in high glucose levels (40 mM) by reducing premature senescence and apoptosis via increased SIRT expression/activity [48]. Metformin has been shown to promote SIRT 1 activity via AMPK pathway. This reduced the oxidative stress caused by hyperglycaemia in a dose-dependent manner [49]. Thus, we speculate that observed effect of metformin on cEPCs in our study may be due to improved cell survival, decreased senescence and/or increased recruitment from the bone marrow. Other beneficial effects of metformin have been achieved, although at very high unphysiological metformin levels only, such as activation of AMPK-mTOR and AMPK-eNOS-NO pathway [50].

EPC mobilisation can be increased via activation of eNOS pathway in diabetes mellitus [51]. Thereby, we can infer that activation of eNOS pathway can increase EPC mobilisation. However, this speculative and needs confirmation using metformin at physiological concentration. For that purpose, we have constructed an angiogenic model to study the mechanism of metformin at physiological concentration. In this experiment, metformin improved angiogenesis through increased angiogenic signal. It not only increased VEGF-A levels but also downregulated angiogenic inhibitors; CXCL-10 and TIMP-1 [52].

Our work can be meaningfully extended by addressing the limitations of our study. Although, there was a small number of patients in this study it was adequately powered. Our type 1 diabetes cohort was heterogeneous with a wide range of diabetes duration and age. This may seem to be a limitation but can also be seen as an advantage to improve generalisability of the results of our study. CGMS was done at the beginning and middle of the study and not at the end of the study. However, CGMS in the middle of the treatment phase was done at the maximum dose of tolerated metformin. Therefore, it is representative of metformin effect on blood glucose levels. We did not use randomised design nor long intervention (8 weeks only). As this research was designed as a proof of concept study exploring the effect of metformin on cEPCs and cECs, data generated from our work can be used to design randomised trials of longer duration in order to repurpose this widely used type 2 diabetes drug, for patients with type 1 diabetes [53]. Our work can be supplemented by exploring the effect of metformin treatment on endothelial function, inflammatory and adhesion markers.

## Conclusions

In summary, our study has shown for the first time that metformin treatment may result in cardiovascular benefit by increasing markers of vascular repair or health (cEPCs, CFU-Hill's colonies, and PACs) and reducing markers of vascular damage (cECs). In a pivotal study by Werner et al. [19], higher levels of cEPCs lead to reduced CVD events. It appears that a 75 % rise in cEPCs number in type 1 diabetes patients as seen in our study might equate to the reclassification of our patients into a lower CVD risk group with approximate Hazard Ratio for CVD death of 0.77 thus 23 % reduction [19]. However, this needs to be confirmed by large randomised controlled trial examining cardiovascular events.

## Additional file

10.1186/s12933-016-0413-6 Correlation matrix of associations between the changes in cEPCs, PACs, PAC adhesion, CFU-Hill colonies and CECs in treatment group. **Table S2**. The univariate and multivariate relationship between changes in cEPC, PACs, CFU-Hill Colonies, CECs and PAC adhesion to changes in other metabolic markers.
