# Supplementary material for: Metformin improves circulating endothelial cells and endothelial progenitor cells in type 1 diabetes: MERIT study
Source: Cardiovasc Diabetol. 2016 Aug 26;15(1):116. doi: 10.1186/s12933-016-0413-6 (PMC5000450; doi:10.1186/s12933-016-0413-6)
Supplement: Supplementary file 1 — 10.1186/s12933-016-0413-6 Correlation matrix of associations between the changes in cEPCs, PACs, PAC adhesion, CFU-Hill colonies and CECs in treatment group. Table S2. The univariate and multivariate relationship between changes in cEPC, PACs, CFU-Hill Colonies, CECs and PAC adhesion to changes in other metabolic markers. [file 12933_2016_413_MOESM1_ESM.docx]

**Supplementary material**

**Methods:**

#### **Proangiogenic cells (PACs)**

PBMNCs were isolated using Ficol protocol. 1 x 10^6^ PBMNCs were plated on fibronectin-coated 24-well culture plate in complete endothelial basal medium (EBM-2; Promo cell supplemented with 20% fetal calf serum and growth factors-hEGF, HC, VEGF, hbFGF, R3IGF, AA and Hep-11). After 48 hours, non-adherent cells were removed. Remaining adherent cells were washed twice with PBS and further incubated for 48 hours. On day 4, adherent cells were stained with acetylated LDL (DiLDL) and Ulex lectin (Sigma) as previously described [1]. Cells staining positive for both Ulex lectin and DiLDL were classed as PACs. 15 random high power fields (200X) were photographed and cells which were dual positive were counted.

#### **Colony Forming Unit (CFU) - Hills’ colonies**

Colony forming unit characterisation has been described before [2]. Briefly, 5 x 10^6^ PBMNC were plated on fibronectin coated 6-well culture plate. After 48 hours, non-adherent cells were collected and plated for seven days on fibronectin coated 24 well plates in the complete endothelial basal medium. On day seven, colonies showing features of spindle-shaped multiple cells originating from a central cluster of round cells were counted in a minimum of three wells and identified as CFU-Hills colonies.

#### **PACs function: fibronectin adhesion assay**

Adherent cells, known as PACs, were detached gently from fibronectin coated 6-well culture plate using 5 mmol/L EDTA [3]. Once detached, these cells were washed in PBS and 1 x 10^5^ cells were re-plated on fibronectin coated 48-well plate in endothelial basal medium (EBM-2; Promo cell) supplemented with 5% fetal calf serum. The cells were incubated for 30 minutes at 37^o^C as previously described [4]. Subsequently, 48-well culture plate was vigorously washed. Remaining adherent cells were stained with LDL (DiLDL) and Ulex lectin as mentioned above. 10 random high power fields (hpf) (200X) were photographed and cells which were dual positive were counted.

**References:**

[1] Vasa M, Fichtlscherer S, Aicher A, et al. (2001) Number and migratory activity of circulating endothelial progenitor cells inversely correlate with risk factors for coronary artery disease. Circulation research 89: E1-7

[2] Hill JM, Zalos G, Halcox JP, et al. (2003) Circulating endothelial progenitor cells, vascular function, and cardiovascular risk. The New England journal of medicine 348: 593-600

[3] Tepper OM, Galiano RD, Capla JM, et al. (2002) Human endothelial progenitor cells from type II diabetics exhibit impaired proliferation, adhesion, and incorporation into vascular structures. Circulation 106: 2781-2786

[4] Huang PH, Chen YH, Chen YL, Wu TC, Chen JW, Lin SJ (2007) Vascular endothelial function and circulating endothelial progenitor cells in patients with cardiac syndrome X. Heart 93: 1064-1070

**Results:**

|  | **cEPCs** | **PAC** | **PAC adhesion** | **CFU-Hill Colonies** | **CECs** |
| --- | --- | --- | --- | --- | --- |
| **cEPCs** |  | 0.153 | 0.05 | -0.25 | 0.13 |
| **PACs** |  |  | 0.47 | 0.41 | -0.26 |
| **FAA** |  |  |  | 0.06 | 0.15 |
| **Hills** |  |  |  |  | -0.6* |

**Table S1:** Correlation matrix of associations between the changes in cEPCs, PACs, PAC adhesion, CFU-Hill colonies and CECs in treatment group. cEPCs: Circulating endothelial progenitor cells CD45^dim^CD34^+^ VEGFR-2^+^ ; PACs: Pro-angiogenic cells; CECs: Circulating endothelial cells. * p=0.003

|  | Univariate analysis | | Multivariate regression analysis | |
| --- | --- | --- | --- | --- |
| cEPC | r | p value | Standardised β Coefficient | p value |
| HbA1c | 0.175 | 0.425 | -0.235 | 0.484 |
| Total cholesterol | -0.13 | 0.544 |  |  |
| Triglyceride | -0.072 | 0.744 |  |  |
| Weight | 0.174 | 0.428 |  |  |
| BMI | 0.293 | 0.175 | 0.451 | 0.442 |
| LDL cholesterol | -0.369 | 0.083 | 0.337 | 0.483 |
| HDL cholesterol | -0.198 | 0.366 |  |  |
| Insulin | -0.244 | 0.262 | 0.170 | 0.64 |
| PAC |  |  |  |  |
| HbA1c | -0.001 | 0.995 | 0.048 | 0.888 |
| Total cholesterol | -0.223 | 0.307 |  |  |
| Triglyceride | -0.641 | 0.001 |  |  |
| Weight | -0.067 | 0.763 |  |  |
| BMI | -0.05 | 0.821 | 0.0476 | 0.417 |
| LDL cholesterol | -0.079 | 0.721 | 0.22 | 0.65 |
| HDL cholesterol | 0.124 | 0.573 |  |  |
| Insulin | 0.085 | 0.701 | -0.064 | 0.861 |
| PAC adhesion |  |  |  |  |
| HbA1c | 0.054 | 0.805 | 0.187 | 0.535 |
| Total cholesterol | -0.229 | 0.292 |  |  |
| Triglyceride | -0.09 | 0.684 |  |  |
| Weight | -0.315 | 0.143 |  |  |
| BMI | -0.216 | 0.322 | -0.199 | 0.368 |
| LDL cholesterol | 0.272 | 0.209 | 0.042 | 0.923 |
| HDL cholesterol | -0.0301 | 0.163 |  |  |
| Insulin | 0.098 | 0.655 | 0.259 | 0.421 |
| CFU-Hill Colony |  |  |  |  |
| HbA1c | 0.224 | 0.304 | 0.187 | 0.535 |
| Total cholesterol | -0.069 | 0.754 |  |  |
| Triglyceride | -0.364 | 0.087 |  |  |
| Weight | -0.101 | 0.647 |  |  |
| BMI | -0.129 | 0.559 | -0.411 | 0.433 |
| LDL cholesterol | 0.034 | 0.878 | 0.042 | 0.923 |
| HDL cholesterol | -0.035 | 0.876 |  |  |
| Insulin | -0.013 | 0.954 | 0.259 | 0.421 |
| CEC |  |  |  |  |
| HbA1c | -0.287 | 0.185 | -0.212 | 0.393 |
| Total cholesterol | -0.286 | 0.185 |  |  |
| Triglyceride | 0.3 | 0.165 |  |  |
| Weight | -0.116 | 0.599 |  |  |
| BMI | -0.12 | 0.584 | -0.263 | 0.464 |
| LDL cholesterol | 0.084 | 0.704 | -0.318 | 0.268 |
| HDL cholesterol | -0.182 | 0.407 |  |  |
| Insulin | -0.345 | 0.107 | -0.294 | 0.166 |

**Table S2:** The univariate and multivariate relationship between changes in cEPC, PACs, CFU-Hill Colonies, CECs and PAC adhesion to changes in other metabolic markers. cEPCs: Circulatory endothelial progenitor cells CD45^dim^CD34^+^ VEGFR-2^+^ ; PACs: Pro-angiogenic cells; CECs: Circulatory endothelial cells.
